# Health Reporting in Print Media in Lebanon: Evidence, Quality and Role in Informing Policymaking

**DOI:** 10.1371/journal.pone.0136435

**Published:** 2015-08-26

**Authors:** Fadi El-Jardali, Lama Bou Karroum, Lamya Bawab, Ola Kdouh, Farah El-Sayed, Hala Rachidi, Malak Makki

**Affiliations:** 1 Department of Health Management and Policy, American University of Beirut, Riad El Solh, Beirut, Lebanon; 2 Knowledge to Policy (K2P) Center, Faculty of Health Sciences, American University of Beirut, Riad El Solh, Beirut, Lebanon; 3 Center for Systematic Reviews on Health Policy and Systems Research (SPARK), American University of Beirut, Riad El Solh, Beirut, Lebanon; 4 Research, Advocacy and Public Policy-making Program, Issam Fares Institute for Public Policy and International Affairs, American University of Beirut, Riad El Solh, Beirut, Lebanon; 5 Department of Clinical Epidemiology and Biostatistics, McMaster University, Hamilton, Ontario, Canada; 6 Assafir newspaper, Beirut, Lebanon; Public Health Agency of Canada, CANADA

## Abstract

**Background:**

Media plays a vital role in shaping public policies and opinions through disseminating health-related information. This study aims at exploring the role of media in informing health policies in Lebanon, identifying the factors influencing health reporting and investigating the role of evidence in health journalism and the quality of health reporting. It also identifies strategies to enhance the use of evidence in health journalism and improve the quality of health reporting.

**Methods:**

Media analysis was conducted to assess the way media reports on health-related issues and the quality of reporting using a quality assessment tool. Semi-structured interviews were also conducted with 27 journalists, researchers and policymakers to explore their perception on the role of media in health policymaking and the factors influencing health reporting. In addition, a validation workshop was conducted.

**Results:**

Out of 1,279 health-related news articles identified, 318 articles used certain type of evidence to report health issues 39.8% of which relied on experts’ opinions as their source of evidence while only 5.9% referenced peer-reviewed research studies. The quality of health reporting was judged to be low based on a quality assessment tool consisting of a set of ten criteria. Journalists raised concerns about issues impeding them from referring to evidence. Journalists also reported difficulties with the investigative health journalism. Policymakers and researchers viewed media as an important tool for evidence-informed health policies, however, serious concerns were voiced in terms of the current practice and capacities.

**Conclusion:**

Our study provides a structured reflection on the role of media and the factors that influence health reporting including context-specific strategies that would enhance the quality and promote the use of evidence in health reporting. In the light of the political changes in many Middle Eastern countries, findings from this study can contribute to redefining the role of media in strengthening health systems.

## Introduction

Media is an important source of health-related information for the public, policymakers and health professionals. It can influence individuals’ health behaviors, prompt policymakers to make decisions to promote health and disseminate health research results to the medical and science community [1–4].

Media plays a vital role in shaping public policies through influencing the different stages of the policymaking process [5]. The Kingdon model (1984) considers the mass media to play a crucial role in opening a policy window where interest groups pinpoint problems, advocate for solutions, and exert pressure on the government to respond [6–8]. However, media’s influence can go beyond agenda-setting; it can influence the whole policy process [9], by speeding up or slowing down the process through highlighting some issues of policymaking more than others [10]. Moreover, through media, policies can be evaluated and policymakers can be held accountable for their decisions [9].

Media can be the conduit that links the public, the civil society and researchers to policymakers. Civil Society Organizations (CSOs) and researchers can use media to promote issues to the forefront of policymakers’ agenda [7, 8]. Furthermore, the media can be used as a strategy to engage the public and the different stakeholders in policy development and implementation. Public engagement in policy development and implementation encourage participative democracy, transparency and accountability [11].

Media can also be a tool to promote evidence-informed policymaking and bridge the existing “know-do” gap through disseminating research results to policymakers and the public [12]. Evidence in the policymaking process consists of information derived from research, feedback from projects and programs, views of the public such as CSOs, expert opinion [13], in addition to experiences from other countries, reports conducted by academic, research and public institutions, international organizations or consultancy companies. However recent studies showed that misreporting evidence provides the public and policymakers with inadequate information, leading to misguided health choices and inappropriate health policies [14–18]. Literature suggests many factors hindering the dissemination and the accurate reporting of health research in the media. These factors include journalists’ lack of time, limited publishable time, lack of science background, difficulties in understanding jargons, complexities of health issues, commercial pressures and problems with finding and using resources [17, 19, 20]. Researchers also worry about the inaccuracy and sensationalism of their work by the media [21–23].

In the Eastern Mediterranean Region (EMR), reporting research evidence in the media is scarce. Newspaper articles in this region are among the lowest in using or describing health systems research and policy dialogues [24]. A study conducted in 12 countries from the EMR showed that researchers considered the media, including newspaper articles, as a strong strategy for promoting the use of evidence in policymaking [25]. Furthermore, policymakers from the EMR reported that the media exerted a strong influence on the health policymaking process in the region [26].

### The Lebanese context

To the best of our knowledge, few studies explored the quality of health reporting in media and the use of evidence in health journalism in the EMR and none in Lebanon. This study explores the quality of health reporting and the use of evidence in health journalism and policymaking in Lebanon. Lebanon is a Low and Middle Income Country (LMIC) in the EMR, where sectarianism plays a major role in the political life. The political parties, represented in the Parliament and in the cabinet, are mainly sectarian-based parties that have a great impact on the political system [27]. Sectarian-based parties also have great influence on the media in Lebanon where most of media outlets including newspapers, televisions and radios are owned by these parties [28]. There are over 20 daily newspapers in Lebanon published in Arabic, English and French which are provided in web versions as well as in print form.

In Lebanon, the health care system is characterized by the multiplicity of financing intermediaries including the NSSF that cover the formal sector of employees, the Civil Servant Cooperative (CSC) that covers civil servants, four military schemes that cover the uniformed armed forces, in addition to the private insurance and the Ministry of Public Health (MOPH) [29]. In addition, Lebanon still lacks a social insurance system that provides health coverage for elderly and workers after retirement and still suffers from weak capacities of public institutions. The study aims to 1) explore the nature of media coverage of health stories and evidence, 2) assess the quality of health reporting, 3) understand the role of media in health policymaking, 4) identify the factors influencing health reporting and 5) identify strategies to enhance the use of evidence in health journalism. Findings from this study will help designing strategies that would enhance the quality of health reporting. Study findings will also inform efforts aiming at strengthening the role of the media in communicating research results to the public and policymakers and in setting policymakers’ agenda in Lebanon and other LMICs in the EMR.

## Methods

The study employed a mix of quantitative and qualitative research design. Data was collected from two sources: 1) media review and 2) key informant interviews and then validated in an interactive workshop.

### Media review

Newspaper articles were analyzed to investigate the role of evidence in health journalism, the way newspaper articles are reporting health stories and evidence and the quality of this reporting. Articles reviewed for this study included articles from Arabic, English and French newspapers (tabloid and broadsheet) published in Lebanon that tackled health-related topics issued between January 2012 and December 2013. The newspaper articles were obtained from the archive databases of the Syndicate of Hospitals and the Ministry of Public Health (MOPH). These two databases are the most comprehensive databases for health-related newspaper articles in Lebanon. They comprise a complete set of health-related articles that appear in all Arabic, English and French newspapers published in Lebanon.

In order to assess the way newspaper articles are reporting research evidence and the quality of the reporting, each article was reviewed and information was extracted onto a coding form developed using Excel spreadsheet. The coding form was adapted from Bubela & Caulfield (2004), Woloshin & Shwartz (2002), Robinson et al. (2013) and Teixeira et al. (2002) [15, 16, 30; 31]. The section for coding general information of the reviewed articles (S1 Coding Form) was adapted from Bubela & Caulfield (2004). This section mainly identifies author’s name, title and date of publication, key topics and nature of headlines (whether optimistic, pessimistic or neutral) [15, 31]. The nature of the headline whether it is optimistic, pessimistic or neutral refers to whether the story reported is a positive (good news), negative (bad news) or neutral story. For example, the headline “Flawed health care system leaves 2 million people at risk” is considered to be pessimistic in that it reports on bad news while the headline “Medical tourism in Lebanon on rise” is considered as optimistic. Headline such as “Medial conference about gastrointestinal system in Nabatieh” and “Conference on low back pain” is considered to be neutral. Exploring whether the media reports on optimistic (good news) versus pessimistic (bad news) stories is important to understand the world of the media and what makes a story newsworthy for journalists. The criteria for judging the quality of health news articles (S2 Coding Form) were mostly adapted from the quality assessment tool used by Robinson et al. (2013) [30]. Framing the problem criterion was adapted from Bubela & Caulfield (2004) [15] and the criterion for reporting on limitations was adapted from Woloshin & Shwartz (2002) [16]. The form was first piloted on 100 newspaper articles by two independent reviewers and slightly modified to ensure validity and reliability of the tool.

The first section of the coding form identified general information of the article (S1 Coding Form). The second section consisted of the quality assessment tool (S2 Coding Form). The quality of the articles was assessed using a set of ten criteria. The first criterion of the quality assessment tool was about the use of evidence. If the first criterion was met the assessment continues by scoring the criteria, if not the assessment stops. In this study, evidence is defined as information derived from research papers, expert opinions, studies, reports and other sources that support (and give proof to/ back up) what the author is reporting in the article. Articles that did not use any evidence included those that reported health-related events such as awareness and health campaigns, health episodes, and seminars. The remaining nine criteria were scored using 0 and 1 grading system to quantify whether the criteria were met or not, except for the “type of evidence used” criteria where articles were classified into three categories A, B or C depending on the type of evidence used and graded 2, 1, and 0 respectively (Table 1). For newspaper articles, falling under categories A and B, information was retrieved and evaluated against the next four criteria. A grade of 1 was given if the article met each of the following criteria: mentioning the title of the study, the author(s)’ name(s), the organization with which the authors are affiliated and the journal title or conference from which the evidence was retrieved. For the remaining four criteria, information was claimed from articles that fell under category A. Research studies, from which the evidence was used in the articles, were retrieved and evaluated against the last four criteria (again assigning a grade of 1 if the criterion was met): location of the research, reporting on limitations of the study, framing the problem and consistency with the findings of the cited study. After evaluating each newspaper article against the set of criteria, the articles that used evidence were given a score over a total of 10. The mean of these scores was then calculated using SPSS version 21.

**Table 1:** Category A, B and C classification.

Category	Grade	Type of evidence
A	2	Peer-reviewed research studies including:
		- Systematic reviews
		- Randomized controlled trials
B	1	Expert opinions that refer to their own knowledge and experience
		Official or policy documents (laws and decrees)
		Grey literature:
		- Reports from public entities (ministries, government agencies,
		- parliamentary committees etc.)
		- Reports from Non-Governmental Organizations (NGOs), Civil Society Organizations (CSOs) and international organizations
C	0	Articles not providing source for the evidence

The coding and the scoring were conducted independently by two members of the research team. Disagreements between the two reviewers were solved by discussion. A third reviewer was consulted when agreement could not be reached.

### Key informant interviews

Key informants interviews aimed at understanding the role of media in health policymaking, identifying the factors influencing health reporting and identifying strategies to enhance the use of evidence in health journalism. Participants were purposively selected based on a sampling frame. The “journalists” category consisted of journalists including reporters, editors-in-chief and section editors involved in reporting health news in print media. The “policymakers” category included senior and middle level policymakers engaged in health policymaking such as officials from the MOPH, members of the parliamentary health committee, and members of health professional associations (including the Order of Pharmacists, Syndicate of Hospitals, Order of Physicians, Order of Nurses, and the National Social Security Fund (NSSF)). The “researchers” category included researchers conducting health research. Additional participants were identified via a respondent-driven technique whereby interviewees suggested potential key informants who could provide additional information. Key informants identified were approached through a targeted email or a phone call explaining the purpose of the study and its benefits. Of the 35 key informants approached, 27 accepted to be interviewed including 9 journalists, 9 policymakers and 9 researchers. Interviews took place from February till March 2014. Semi-structured face-to-face interviews were conducted and lasted between 45 and 60 minutes. The interviews were transcribed verbatim. Arabic transcripts were translated to English then back-translated to Arabic to ensure the accuracy of the translation process. The participants had diverse backgrounds and experiences to ensure diversity and comprehensiveness of claims.

Three different interview tools were used with each of the journalists, policymakers and researchers (S1 Interview Tool). The Crewe & Young (2002) framework for understanding research-policy links was used as a guide for developing the interview tools. The framework aggregates issues around four areas: *The political context*, *the evidence*, *links and external influences* [32]. The interview tools were piloted with one journalist, one policymaker, and one researcher. They covered the following main topics: role of media in policymaking and public opinion, factors influencing health reporting, factors influencing the use of evidence in health journalism, role of evidence in health journalism, quality of health reporting, and strategies to improve the quality of health reporting and to incorporate evidence into health journalism.

### Validation workshop

An interactive workshop was conducted to validate the findings and receive feedback and insights from the participants. The workshop included 10 journalists that report on health issues from various Lebanese newspapers, news websites, radio and Television. Journalists participating from newspapers were also interviewed during the key informant interviews. The findings of the media review were presented during the workshop where the research team asked the participants’ feedback on what are the factors that affected the quality of the reporting and why there is low proportion of news articles reporting on evidence. They also deliberated on the outcome of the interview findings and discussed the role of media in health policymaking and presented recommendations and strategies to enhance the use of evidence in health reporting. Field notes were taken during the workshop. Field notes were then compared with the thematic analysis of the interviews so information is added or validated where appropriate.

### Ethical considerations

The Institutional Review Board (IRB) at the American University of Beirut granted ethics approval for the study. Interviewees were contacted over the phone or via email to request their participation. Upon their approval and prior to data collection, written informed consent was collected. Interviews were audio-recorded upon request; two interviewees refused to be tape-recorded so their responses were recorded by extensive note-taking. Interviews took place in the interviewees’ workplace based on their requests. The interviews were conducted in Arabic or English depending on the interviewee’s preference. The interview transcripts were anonymous and recordings were immediately destroyed following transcription. All transcripts were reviewed and validated by three members of the research team.

### Data analysis

Qualitative thematic analysis of the interviews was conducted. The responses of all informants were coded using an Excel spreadsheet. Open coding was conducted for each of the three participant categories. The responses were segregated into sections that relate similar concepts. Thereafter, research members discussed and cross-checked the emerging concepts. This was followed by axial coding whereby concepts were organized into themes and subthemes that reflect the study’s objectives.

Data was obtained from both the media review and the key informant interviews to ensure data triangulation. This helped ensure completeness of data collected through the use of different data sources for the same study [33]. The findings from the media review and key informant interviews were further validated through the workshop.

## Results

Findings are presented in two parts. The first part consists of the findings of the media review. The second part presents interview findings on the nature of health journalism, quality of health journalism, the influence of media and research on health policymaking and strategies to enhance the use of evidence in health journalism.

### I. Results from the media review

**Fig 1:**
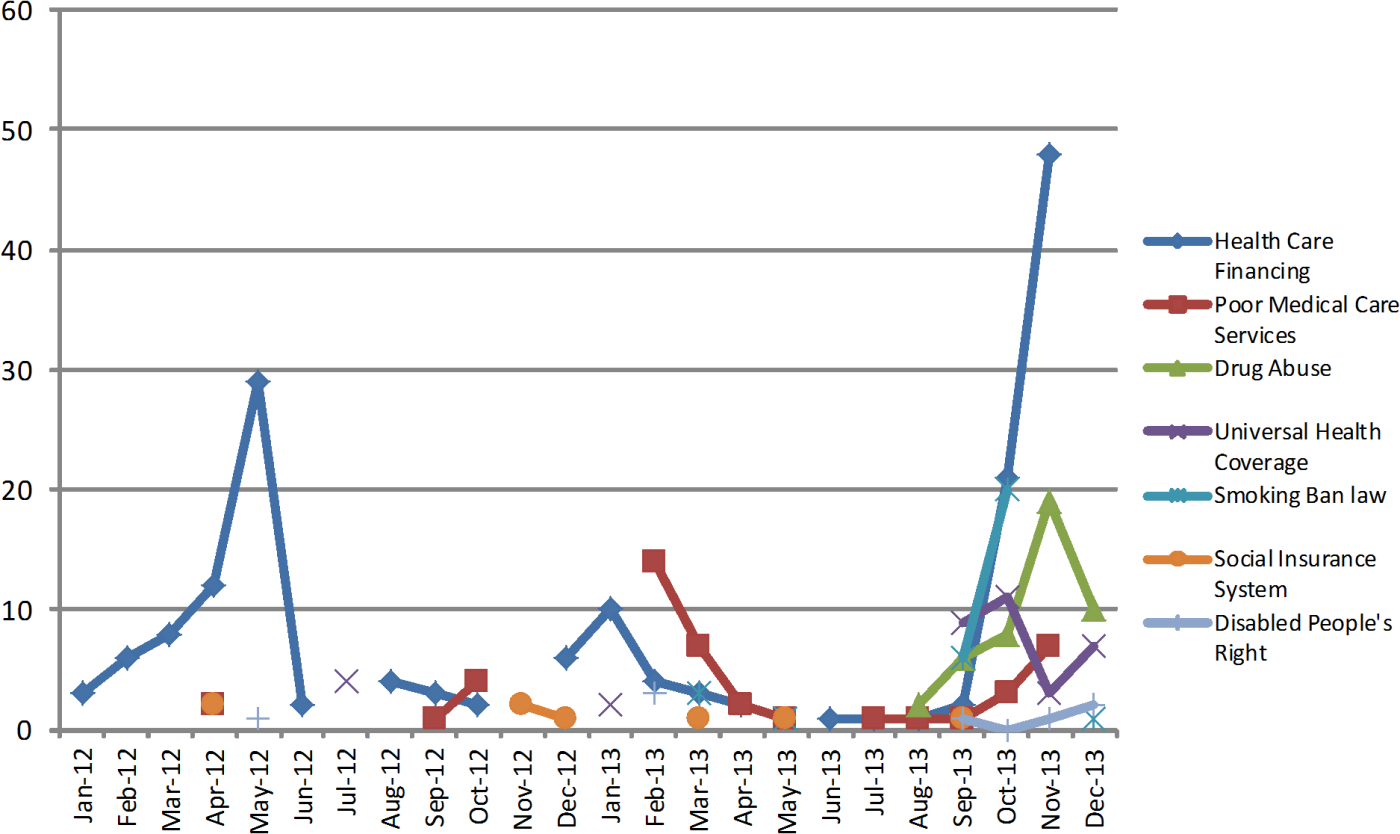
Most recurring topics monthly appearance.

A total of 1,279 newspaper articles reporting health issues were reviewed. The agreement between reviewers was 81%. Seven key health topics recurred in the retrieved articles (Fig 1). The most repeated topic was *health care financing*, reporting the MOPH’s and NSSF’s inability to reimburse hospitals for their claims, this topic appeared at least once every month in 2012 and several times at the end of 2013. The second most recurrent topic was *poor medical care services*, which involved news about patients’ death, admission, safety and medical errors and appeared every other month throughout the study period. The *drug abuse* and *universal health coverage* subjects appeared at the end of 2013. Whereas, the delay of the implementation of the smoking ban law (*law no*. *174)* topic emerged in the newspapers starting March 2013 until the end of the year 27 times. Other topics that appeared were *social insurance system* as a plan under study and *disabled people’s rights*. Although these topics recurred frequently, single incidents were reported at their occurrence and were rarely followed up.

Out of 1,279 articles, 625 (48.8%) incurred optimistic claims, reporting news that implemented more favorable events to the reader, such as opening new clinics and hospital units, fundraisings, awareness campaigns and others. Whereas, 485 (37.9%) incurred pessimistic claims related to less favorable events in Lebanon such as medical errors, deaths and financial crisis. The remaining articles reported either claims that were neutral or articles that had both pessimistic and optimistic claims.

**Fig 2:**
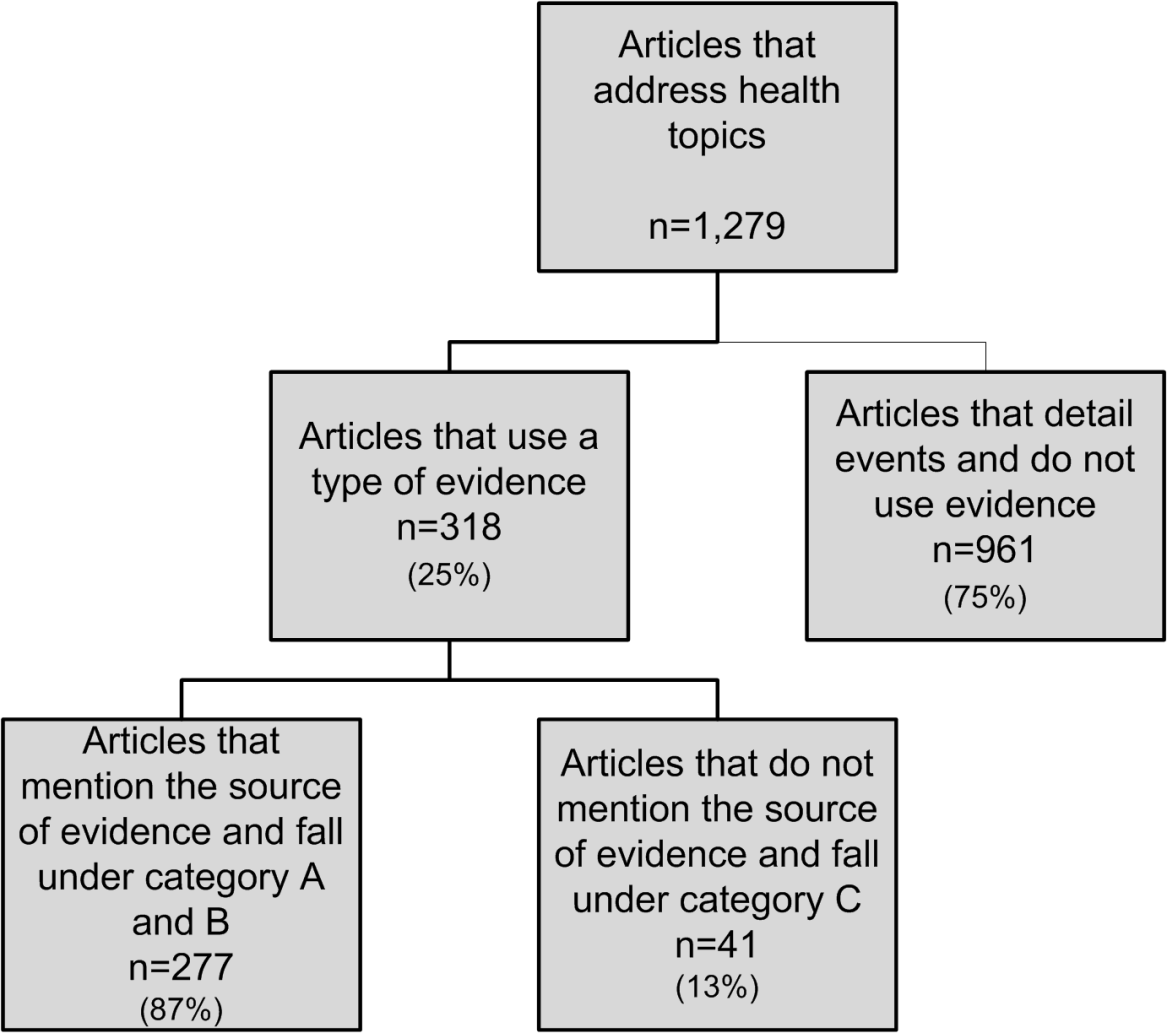
Evidence use in newspaper articles.

The majority of articles (75%) detailed health-related events and did not use evidence, while only a quarter was informative articles that relied on evidence (Fig 2).

**Fig 3:**
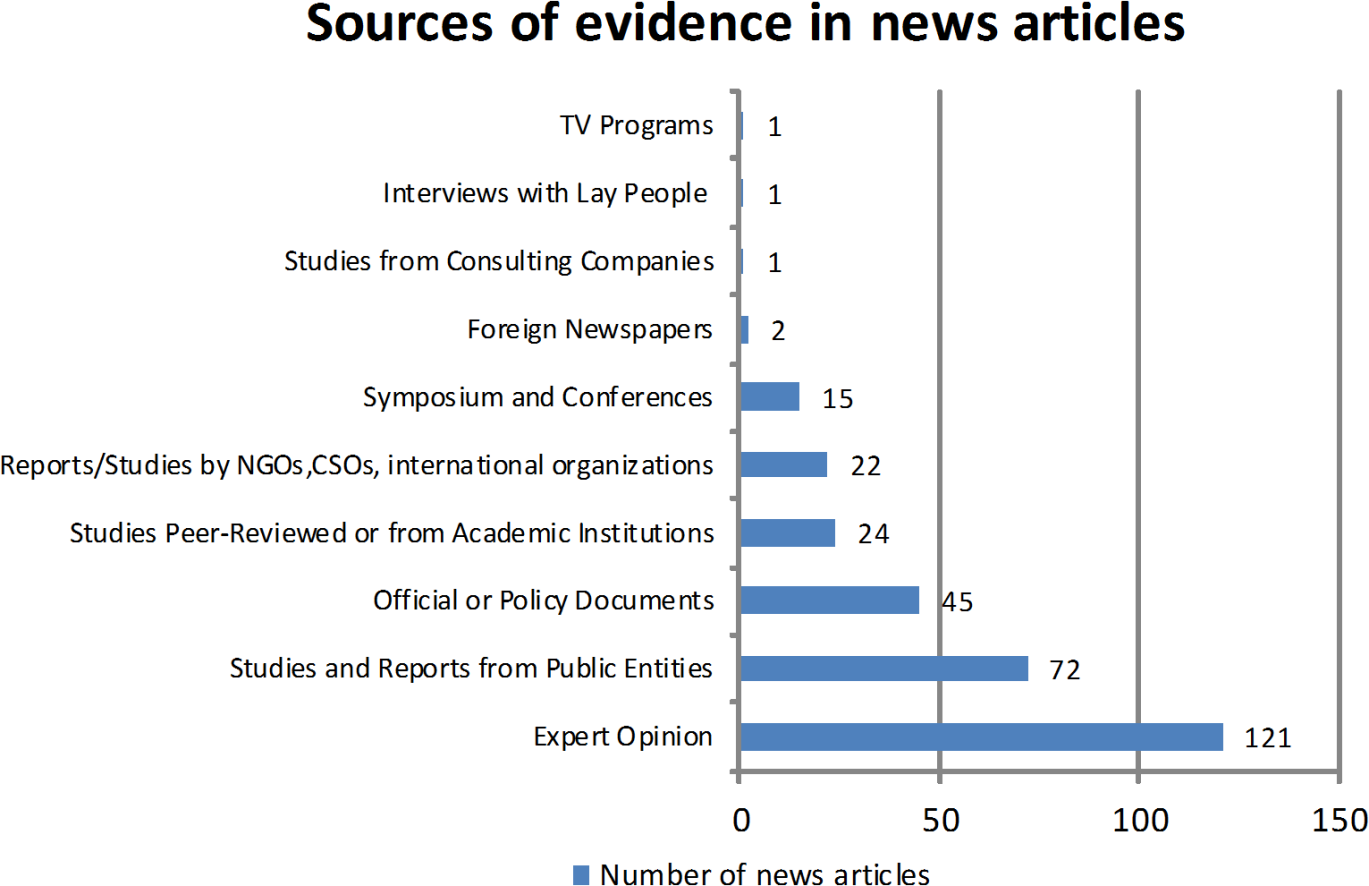
Sources of evidence in news articles.

The 277 newspaper articles that mentioned the source of evidence were grouped into ten different categories based on the source of evidence (Fig 3). These articles might have used more than one source of evidence resulting in a total of 304 sources used in the 272 newspaper articles using evidence. The source that journalists mostly relied on was expert opinion, which was used 121 times (39.8%) out of the total 304 sources. The second commonly used source for health reporting was studies and reports conducted by public entities, which was used 72 (23.6%) times. These were followed by official or policy documents (14.8%), peer-reviewed studies and studies from academic institutions (7.8%) (Fig 3).

#### Quality of newspaper articles reporting evidence

Assessment of the quality of the 318 newspaper articles using evidence resulted in an average quality score of 2.68 (± 1.29) out of a maximum score of 10, with scores ranging from 0 to 8.

**Fig 4:**
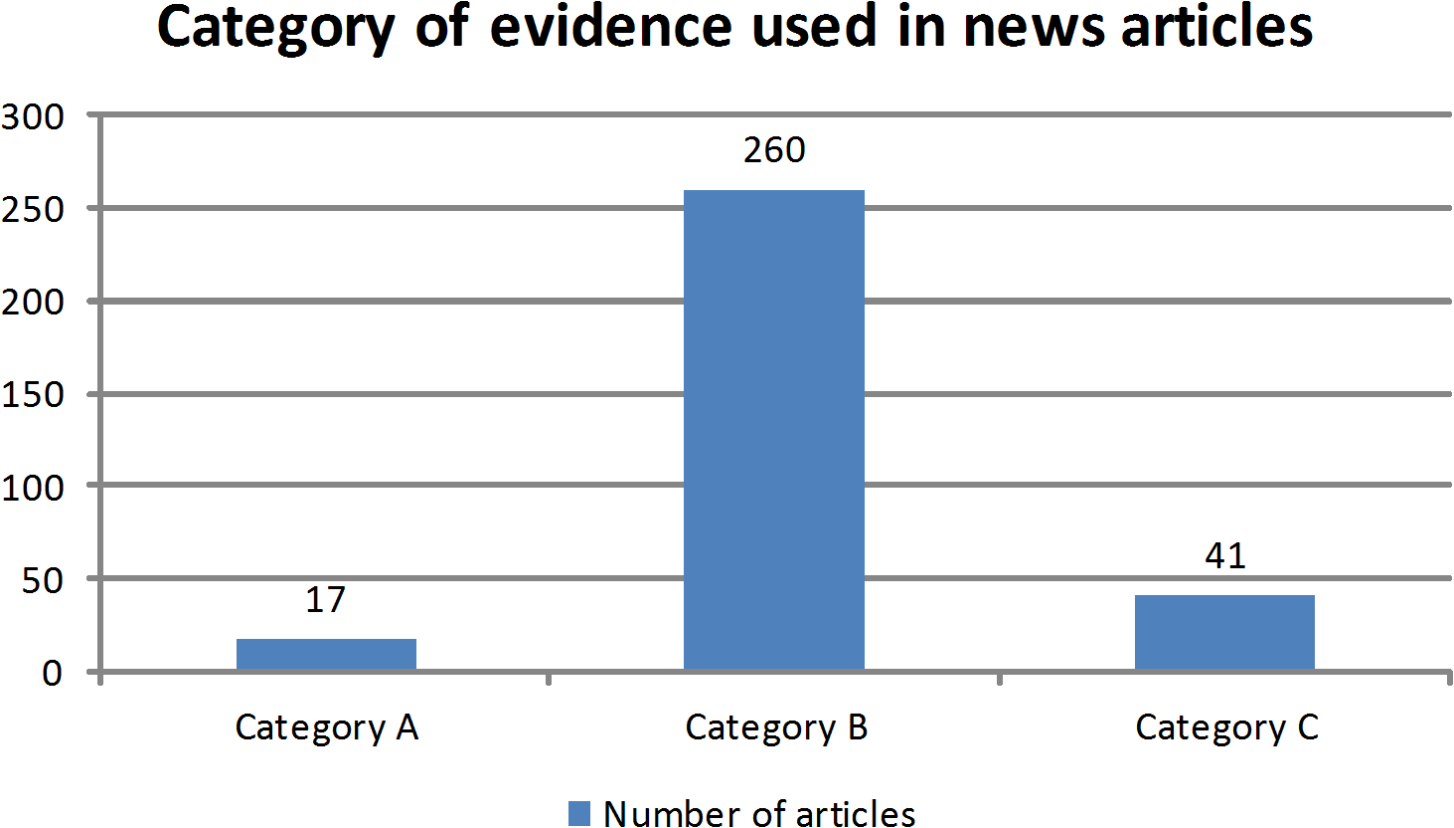
Category of evidence used in newspaper articles.

Articles mostly used evidence of category B (81.8%) followed by category C (12.9%) then A (5.3%) (Fig 4). From the 277 articles under categories A and B, 81 (29.2%) stated the journal which published the study, such as the International Journal of Public Health or the New England Journal of Medicine, or the conference that discussed the evidence, such as the ESMO 2012 conference. Out of the articles under categories A and B, 21 (7.6%) mentioned the name of the author of the study, whereas 119 (42.9%) articles included the organization that the study or the author was affiliated to and two (0.7%) articles mentioned the title of the study. From the 17 articles under category A, that are peer-reviewed or from academic institutions, such as the American University of Beirut or the Lebanese University, 13 articles were consistent with the original study reporting the exact information present in the original study and fifteen reported evidence neutrally as it was reported in the original study without framing the problem into a positive or negative claim. All 17 articles mentioned the location of the research, but none reported on the limitations of the study they referred to.

### II. Results from the key informant interviews

Thematic analysis identified five main themes: nature of health journalism, factors affecting quality of health journalism, evidence use in health journalism,the influence of media and research on health policymaking and strategies to enhance the use of evidence in policymaking.

#### 1. Nature of health journalism

Most journalists emphasized that health is not a priority in Lebanon, and that health journalism is still not well-established, as evident by the lack of daily health sections in the newspapers. One journalist attributed the lack of interest in health journalism to the overall culture. The need for the media to direct its attention more frequently to health issues was mentioned by a number of policymakers.

*“The problem isn’t from policymakers as to why health is not a priority*, *it’s everyone*. *Media doesn’t shed a spotlight on such issues; it doesn’t place it on its first pages*. *Researchers don’t think that these issues should reach the people; they think it’s hard for people to understand*.*”-* A journalist

Interviews with journalists identified four types of health journalism in Lebanon. The first type is related to reporting health research; it is usually disease-oriented and has the goal of educating the public. The second type is related to covering health events including conferences, campaigns and health days. The third type of health journalism is related to covering topics about the health system, whereby journalists use research evidence to back up claims or evaluate the situation. The fourth type of health journalism is investigative journalism, whereby journalists follow up on news and investigate its accuracy, such as news related to the spread of low quality vaccines in the Lebanese market. Most journalists reported several difficulties with the investigative type of health journalism. They reported feeling threatened when working on investigative reports and that they preferred to cover neutral issues instead. As one journalist mentioned:

*“Sometimes*, *I feel threatened because I have heard stories*, *particularly with journalists engaged in investigative journalism where they were exposed to harm for investigating a certain health issue and promoting political action and attempting to provoke changes in policies”*

Most journalists expressed their tendency to report on humanitarian issues, such as violence against women, death due to some disease or the MOPH inability to provide medicine to the less fortunate. The main factors that reportedly influenced journalists’ choice of topics included the timeliness of the topic and journalists’ preferences for and curiosity about certain topics. Most journalists, in both the interviews and the workshop, also highlighted the influence of public relations and advertisement including those by pharmaceutical companies on their choice of topics. At the same time, they all ensured that they stayed away from advertising and promoting products in their reports.

*“A large chunk of media has an advertisement rather than an informative attribute*.*”-* A journalist

Most respondents, including journalists, agreed that journalists report news according to the public’s interests and benefits in order to get high ratings. They also perceived that people are interested in humanitarian news, as previously mentioned, and tabloids rather than health topics and research evidence.

#### 2. Factors affecting quality of health reporting

There are several shortcomings in the quality of health reporting in Lebanon. Almost all journalists disclosed that they judge the credibility of a source of health information based on the source’s professional title, such as whether the source is a physician or a ministry official. This finding was also confirmed in the workshop. Only one journalist mentioned peer-reviewed journals as a trustworthy source of health information.

Main factors affecting quality of health reporting included time and competition among journalists. In the workshop, lack of time was mentioned as a major barrier to constrain journalists from reporting on scientific research evidence. Most journalists admitted that competition and reporting on a scoop might force them to publish articles before being able to get the full information on the news, which in turn might have contributed to policymakers’ and researchers’ loss of trust in the credibility of media. Journalists further justified that they can publish more information after they get the initial news out.

*“If I know about something and I need to write it on the same day*, *I won’t be able to get all the facts*, *because if someone else picks it up [repots on the scoop]*, *there will be no point in my writing about it the next day*.*”*- A journalist

The majority of policymakers and researchers also added that journalists’ inaccuracy in reporting is affecting the quality of health journalism. They stated that the media should intensify its role as an accountability tool for information, especially since people have a tendency to take media reports as facts without further inquiry or examination.

*“The public is affected by the media and tends to believe falsely reported news”*—A policymaker

One main issue that most respondents from all three categories agreed on was that media does not follow-up on news that it has previously reported on.

*“The media never follows up [on news] even if it brings up important issues”*- A policymaker

Another issue that almost all respondents in the interviews and the workshop agreed on is journalists’ lack of specialization in health journalism. Although some of the interviewed journalists had backgrounds in science such as biology, medical laboratory and medicine, still none were specialized in health journalism.

#### 3. Evidence use in health journalism

Most respondents agreed that media does not rely on evidence. This might be due, according to one researcher and one journalist, to the fact that the concept of evidence use and its importance in reporting is still lacking in Lebanon. However, journalists acknowledged the importance of evidence-informed health journalism and of incorporating it into their work routine in order to make their reports more reliable and comprehensive.

*“Time is definitely an issue*, *but I do it [access and read research] anyway because I care about scientific reporting*. *I incorporate it into my work routine*. *It is something important and offers something to the reader and gets us readers as well*.*”-* A journalist

Almost all journalists in the interviews and the workshop preferred interviewing experts to reading research. They explained that they preferred to talk to the researchers and have them explain in brief the aim and results of the study. This was attributed to time limitations, having to work in more than one section and the complexity of research language, which was perceived by some of the journalists as being dry, boring and difficult to translate and simplify to the reader. Many reporters were not aware of the availability of research databases and all save one journalist expressed an intention to access research. Additionally, journalists in the workshop acknowledged that they do not have the skills or institutional capacity to access databases.

*“I usually access information through personal relationships with doctors who inform me of recent health issues and discoveries*. *It is even better when the doctor or researcher conveys the information in the language in which I write in*.*”-* A journalist

Moreover, most of the journalists and one researcher indicated that journalists prefer the use of international rather than local evidence, acknowledging the fact that local research is scarce.

#### 4. Media and research influence on health policymaking

Most journalists agreed that they should be involved throughout the stages of the policymaking process. In the workshop, journalists agreed on the influential role that they may play in the process. However, they acknowledged that extensive and ongoing coverage of health policymaking is difficult to achieve due to time restrictions. Additionally, journalists reported that they felt discouraged from reporting on health policymaking. They explained that they were notified of decisions after a policy is made or implemented mainly because of their limited interaction with policymakers.

*“No one discusses policy issues beforehand*. *We rarely know what’s going on during [policy] development*. *Usually*, *policies are be already implemented then you go and cover it”-* A journalist

Further contributing to their feeling of discouragement is journalists’ perception that their health-related reports have limited implications on policy actions.

*“I feel like whatever I report or cover*, *it does not get results*. *It’s like working for nothing*. *We see no real change in policies and we feel like we are not heard”-* A journalist

Some journalists also mentioned that their inability to measure the impact of their news reports on policymaking was mainly due to the long term nature of this impact and the complexity of such measurement. Additionally, even if evaluation were conducted it would strictly be based on opinions and impressions rather than on evidence.

*“It is difficult to measure that [impact]*. *You can gauge the response of officials by their feedback*. *It’s hard to tell that this is a result of your story”-* A journalist

Moreover, most respondents and journalists from the workshop acknowledged the influential role research can play in informing policymaking, which has yet to be realized in Lebanon. Researchers attributed the gap in the uptake of research to the misalignment of research priorities with those of policymakers. Researchers’ main focus is on publishing research studies in academically valued peer-reviewed journals rather than on disseminating research to policymakers and stakeholders.

*“Publishing in peer-reviewed journals*, *that’s great for our personal and professional development*, *but it’s not effective in policymaking”*—A researcher

Most journalists and researchers and one policymaker alluded to the unstructured nature of the policymaking process in Lebanon. According to them, policy decisions arise as a reaction to surfacing health issues and not as a product of careful planning and agenda setting.

“*Policymaking comes as a reaction*, *not something that is pre-planned or part of an agenda that is being monitored or worked on regularly*”—A researcher

Most journalists and researchers agreed that policymakers are primarily concerned with politics rather than policies rendering them unresponsive to evidence and media reports. Moreover, these respondents perceived policymaking as governed by political and religious interests rather than evidence.

**“** *Policymakers [in Lebanon] are not technocrats*, *they are politicized*. *So they tend to address those issues on an ad-hoc basis*. *They are reactive and only respond to things when they reach crisis proportions*”—A journalist

Policymakers and researchers ascribed media’s narrow impact on policy actions to journalists’ insufficient coverage of health policy issues and their lack of follow up. They also alluded to the absence of systematic framework enabling media to consistently link researchers and policymakers. As one policymaker stated:

“Not once did the media in Lebanon alert me to something useful in my domain as in something that would help me make pertinent decisions”

#### 5. Strategies to enhance the use of evidence in health journalism

Three main strategies to improve the quality of health reporting and the use of evidence in health journalism and policymaking were identified from the key informant interviews and the validation workshop. These strategies are: a) specialization of journalists in health reporting, b) research dissemination through media outlets, academic institutions, journalists’ education and research briefs, and c) forming a platform as a link between media, policymakers and researchers.

The first strategy identified by respondents to enhance the use of evidence in health journalism is the “specialization of journalists into health reporting”.

All respondents agreed on the need for qualified and specialized health journalists, as a means for promoting the use of evidence in reporting.

*“We need specialized journalists and specialized publications*. *Anyone who wants to report on the health sector should have the knowledge and specialty to do so*. *Media specialized in health can play a role in making research reach policymakers”*—A policymaker

Specialization may contribute to establishing health sections within newspapers, which exclusively report health news, and qualifies journalists in health reporting. Some researchers and journalists from the workshop also suggested incorporating health into the curriculum of media specialties. Respondents perceived that such specialization could enhance media’s access to research evidence as well as its understanding and accurate reporting, which could then be channeled into policies.

In addition, as a first step towards specialization, most researchers stressed the importance of training journalists who are currently practicing health reporting. This can be done through educating them on the importance of research evidence as well as providing them with the means to access reliable evidence through databases and interpreting research findings. Most journalists agreed on the importance of training, while keeping in mind their time limitations for such trainings.

The second strategy is “research dissemination to media”. According to most researchers and journalists, academic institutions play an important role in disseminating research to media particularly through university media offices. These offices can attract attention to particular issues and promote public’s awareness of critical health and policy matters. However, some researchers mentioned that institutions do not consider communication with media as a priority, which is reflected in the deficiency of funding media offices and the poor attempts researchers make to reach out to the media.

*“The media office in our faculty hasn't been as responsive as I would have liked them to be and there might be different reasons for that*. *I think the values and the priorities of the people in charge and what they are concerned with in terms of image*, *are from my point of view distorted”-* A researcher

Another suggestion by most of the journalists and researchers is the need for simplified research formats to render research evidence more accessible to journalists and facilitate its use in health reporting. Most journalists, in the interviews and at the workshop, expressed their preference for brief summaries of research that include quantified results, take home messages, public health significance and do not dwell on methodology and technicalities. Journalists preferred research summaries written in a contextualized manner using the same language in which journalists write their reports. They also stressed on the importance of delivering research briefs in a timely manner.

*“I want a summary of the results*, *the main take home ideas and numbers*. *It should be framed in a way that relates to the health benefits of the reader and does not dwell on methods and technicalities”-* A journalist

The third strategy is “forming a platform”. Respondents collectively emphasized that in order for the media to achieve a positive influence on the public and policymaking, it necessitates a well-established network that facilitates communication between journalists, policymakers, and researchers. According to most researchers and journalists, such networks can bridge the gap between the media and the research worlds, promote the use of evidence in health journalism and improve the quality of health reporting.

Connections between researchers and policymakers can also reportedly help establish ongoing dialogues and align researchers’ priorities with those of policymakers. Moreover, some researchers viewed their contact with policymakers as an opportunity to contextualize their research findings.

*“If research is to feed into policy*, *the researcher must understand the needs of the policymakers and their intents to change*, *or have a good knowledge of the context and try to bring in through research new ways to influence policies”-* A researcher

## Discussion

The study findings suggest that the timeliness of a story, the preferences of the journalists and their curiosity about certain issues influence the newsworthiness of a story. The nature of the news claim, whether pessimistic or optimistic, has a lesser influence since optimistic stories were almost equally reported as the pessimistic ones. This contradicts with the findings of previous studies that showed that journalists tend to report bad news which might be appealing to the readers. For example, a study conducted by Bartlett et al. (2002) showed that pessimistic news are more likely to be reported than optimistic news [34]. Our study also pointed out the limited follow-up on news by the journalists and the difficulties in practicing investigative journalism. This is supported by the findings of the media review that shows that single incidents were rarely followed up.

### Quality of Health Reporting

Results from the media review, the key informant interviews, and the validation workshop confirmed the low quality of health reporting and the limited use of evidence in health reporting. Respondents reported that evidence is not often used in media, which affects the quality of reporting. Interview findings suggest many factors behind this limited use of evidence which include time constraints, the complexity of research language, competition, lack of specialization in health journalism, the limited skills and institutional capacity to access databases and the scarcity of local research. This claim was further supported by the limited number of news articles that use evidence and the low quality of reporting obtained from the media analysis. The media analysis showed that the low quality of health reporting is mainly attributed to the failure of journalists to report on the limitations of the studies and to mention the source of evidence, the title of the study and the author’s name. Another main reason behind the low quality of health reporting is the reliance on expert opinion as the main source of evidence in health reporting. Expert opinions were found, by the media analysis, to be the most frequently used source of evidence in health reporting, while the use of peer-reviewed studies was found to be scarce, yet again affecting the quality of reporting. Similarly, Albaek (2011) reported that the use of expert opinions as a source of evidence in journalism is on the rise [35]. This may be attributed to journalists’ perception of research as dry and boring, in addition to difficulties and time limitations to access, understand and translate research studies. Similar findings were reported by Voss (2002), Larsson et al., (2003) and Ashorkhani et al., (2012) linking difficulties in evidence based health reporting to time pressures and the rigid nature of evidence [17,19,20]. The limited use of evidence in health reporting was also found in two international health information television programs as reported in Korownyk et al. (2014). Around half of the recommendations lacked evidence or are contradicted by evidence [36]. Robinson et al (2013) indicated that the higher the level of evidence used in health reporting the higher the quality of the newspaper article [30].

### Lack of specialized health journalists

One major finding was the lack of health-specialized journalists as well as the lack of sections dedicated to health in newspapers in Lebanon. Respondents from the interviews showed the need for specialized health journalists, as a means for enhancing the quality of reporting and promoting the use of evidence in reporting which could then be channeled into policies. The lack of specialized journalism was also linked to low quality of health reporting in Iran [17]. Similarly, a study from Australia found higher quality of stories written by specialized health journalists compared to those written by non-specialists in the same media outlet [37].

### Media and health policymaking

The interviews findings highlighted the limited interactions between policymakers and journalist which have led to discouragement of journalist from reporting on health policymaking. In addition, policymakers and researchers expressed their lack of trust in the credibility and accuracy of media reporting. This lack of trust in health journalism was also established in other studies whereby media was found to be incapable of conveying research messages accurately to the public [18, 38]. Other reported barriers impeding the interaction between journalists from one side and policymakers and researchers from other side include poor communication skills of researchers and lack of alignment of priorities between these groups. These findings were supported in other studies from the EMR, whereby the policymakers mainly rely on politics and opinions rather than on evidence and researchers’ main incentives were primarily to publish in peer reviewed journals [25, 26, 38]. Moreover, the misalignment between media and researchers’ priorities highlighted in this study was also revealed in a study from Canada, where journalists were found to rely on researchers to help them interpret events, and researchers are rarely available and have little patience when it comes to giving explanations to journalists [39].

This study has highlighted concerns from policymakers, journalists and researchers of the limited impact of the media on policymaking. This is in line with findings from Cook et al. (1983), where media is capable to inform the public but not to generate policy action [2]. Moreover, a recent print media analysis in LMICs, including Lebanon, concluded that the climate for evidence-based policymaking in those countries is still premature [24].

### Strategies to enhance the use of evidence in health journalism

This study identifies three main strategies to improve the quality of health reporting and the use of evidence in health journalism based on the findings of the key informant interviews and the validation workshop. These strategies are specialization of journalists in health reporting, research dissemination through media outlets, academic institutions, journalists’ education and research briefs, and forming a platform as a link between media, policymakers and researchers.

Study findings on strategies to enhance the use of evidence in health journalism concur with the findings of Oxman et al. (2009) that suggests strategies to ameliorate to role of media in engaging the public in evidence-informed policymaking [11]. One of the strategies identified by the respondents is to simplify the research format presented to the media, which comes in line with literature highlighting the importance of media releases in facilitating access of journalists to evidence [11,17]. Journalists interviewed conceptualized their preferred format of the research summary as brief, simple and result-oriented. Developing summaries with policy recommendations for decisions-makers was also found to be a facilitator to the use of research evidence in health policymaking in a study from the EMR [39]. Another suggestion was to educate and train health journalists on the importance and use of evidence. A study by Schwitzer et al. (2005) revealed the effectiveness of educating journalists to facilitate evidence based health reporting [40]. In their study Oxman et al. (2009) found that training journalist enhanced the accuracy and hence the quality of health reporting [11]. Moreover, all stakeholders interviewed emphasized the need to establish networks to govern the ongoing interaction and dialogue amongst them. Literature shows that such an interaction increases the chances of mediating research to policymakers for promoting evidence-informed policymaking [41–43]. A qualitative study identified building relationships between researchers and journalists as an effective strategy for the cultivation of an environment supporting evidence based health reporting [44].

### Strengths and Limitations

This study has several strengths. First, the study is the first of its kind in the EMR to explore the quality of health reporting in media and the use of evidence in health journalism and policymaking. Second, the study employed data triangulation to ensure completeness and comprehensiveness of the data collected from both the media review and the key informant interviews, in addition to data validation by conducting a workshop. Third, the key informant interviews involved a diverse sample of journalists, researchers and policymakers with various backgrounds, which helped provide in depth views on the use of evidence in health reporting. Finally, the study provides strategies that can be used to guide future action in the EMR and in LMICs for promoting evidence use in health reporting and policymaking.

This study has a few limitations. First, data for the media review was only collected from print media thus health news articles published online via news websites or social media, and health news broadcasted on television were not assessed. However we can assume that print media can be representative of the other sources of evidence since the findings were validated by the workshop that was attended by journalist from all media means (print, television, radio, news websites). Second, 9 of the 16 journalists approached accepted to be interviewed which means we did not capture all the journalists. However, we believe that those journalists have fully represented the experiences and perception of this category of participants and we have reached data saturation. Third, social desirability bias is a second limitation that might have influenced interview responses. Respondents may have provided the answers they considered desirable by the investigators. However, it can be safely assumed that the results are not overly inflated as findings were cross-checked with those from the media review and the workshop. Finally, recall bias might be one of the limitations. However, recall bias had minimal influence on study results since findings were validated by the media review and the validation workshop.

### Implications for Policy and Practice

In light of the changes in the EMR and the transition towards democratic systems, findings from this study can contribute to the efforts of strengthening and redefining the role of media in this region. Media can play a greater role in informing the public, advocating for policy changes and impacting public policymaking especially during this period as new political systems are being built in the region. Media can empower citizens groups and promote community participation in policymaking as well as in planning and monitoring health services. Findings from this study can also help provide ways for moving forward for improving the use of evidence in health journalism mainly by focusing on strengthening the relationship between journalists, researchers and policymakers and building the capacity of journalists in evidence-based health reporting. This will reinforce the role of the media in the EMR, and increase its reliability as a source of research evidence to be able to influence policy and policymaking, while strengthening its role as an accountability tool.

## Conclusion

This study sheds the light on the lack of appropriate use of research evidence in health journalism, the low quality of health reporting in newspaper articles and the lack of specialization in health journalism in Lebanon. The gap between the media and research world is still wide. Journalists reported many factors hindering the use of research evidence in health reports mainly time constraints and the complexity of research language and the lack of specialization in health journalism. Policymakers also showed concerns regarding the limited role of media in influencing the public and policymakers’ decisions. This study suggests strategies that can improve the use of evidence in health reporting. The findings of this study would also contribute to designing context-specific knowledge translation strategies that would enhance the role of media in channeling evidence into policies.

## Supporting Information

S1 Coding Form General information about the health news articles. (DOCX)

S2 Coding Form Criteria to judge the quality of health news article. (DOCX)

S1 Data Abstraction Form (XLS)

S1 Interview Tool (DOCX)
