# Supplementary material for: Health Reporting in Print Media in Lebanon: Evidence, Quality and Role in Informing Policymaking
Source: PLoS One. 2015 Aug 26;10(8):e0136435. doi: 10.1371/journal.pone.0136435 (PMC4550456; doi:10.1371/journal.pone.0136435)
Supplement: S1 Coding Form — (DOCX) [file pone.0136435.s001.docx]

**Coding Form S1**

**General information about the health news articles**

| Criteria | Description | |
| --- | --- | --- |
| Name of Author |  |  |
| Title of Publication |  | |
| Source of Publication |  | |
| Language |  | |
| Date of Publication |  | |
| How did you find this article? |  | |
| Link to Article (if the article was retrieved from a webpage) |  | |
| Key topics |  | |
| Optimistic, pessimistic, neutral claim |  | |
