# Supplementary material for: Health Reporting in Print Media in Lebanon: Evidence, Quality and Role in Informing Policymaking
Source: PLoS One. 2015 Aug 26;10(8):e0136435. doi: 10.1371/journal.pone.0136435 (PMC4550456; doi:10.1371/journal.pone.0136435)
Supplement: S2 Coding Form — (DOCX) [file pone.0136435.s002.docx]

**Coding Form**

| Criteria | Description | Score |
| --- | --- | --- |
| 1 | Use of evidence: Is the article using evidence? | Yes=1 No=0 |
| 2 | Source of evidence: Does the news article mention the source of the evidence? | Yes=1 No=0 |
| 3 | The type of evidence used: What is the type of evidence used? | Grade A=2 Grade B=1 Grade C=0 |
| 4 | Title of the study: Does the news article mention the study title? | Yes=1 No=0 |
| 5 | Author(s)’ name(s): Does it mention the author(s)’ name(s)? | Yes=1 No=0 |
| 6 | Affiliated organization: Does it mention the affiliated organization? | Yes=1 No=0 |
| 7 | Location of the research: Does the location of the research? | Yes=1 No=0 |
| 8 | Consistency with study findings: Are the findings reported consistent with the study findings? | Yes=1 No=0 |
| 9 | Framing of the problem: Is the story reported neutrally and not framed negatively or positively? | Yes=1 No=0 |
| 10 | Reporting on limitations: Does the news article report limitations of the study? | Yes=1 No=0 |

**Criteria to judge the quality of health news article^[[1]](#footnote-1)^**

1. Adapted from:

   Robinson A., Coutinho A., Bryden A., McKee M. 2013. Analysis of health stories in daily newspapers in the UK. *Public Health*, 127:39-45.

   Bubela, T.M. and T.A. Caulfield. (2004). “Do the Print Media ‘Hype’ Genetic Research? A Comparison of Newspaper Stories and Peer-Reviewed Research Papers.” *Canadian Medical Association Journal* 170: 1399–407.

   Woloshin S. & Shwartz L. M. 2002. Press Releases: Translation research into news. *JAMA*; 287 (21):2856-8. [↑](#footnote-ref-1)
