# Supplementary material for: Health Reporting in Print Media in Lebanon: Evidence, Quality and Role in Informing Policymaking
Source: PLoS One. 2015 Aug 26;10(8):e0136435. doi: 10.1371/journal.pone.0136435 (PMC4550456; doi:10.1371/journal.pone.0136435)
Supplement: S1 Interview Tool — (DOCX) [file pone.0136435.s004.docx]

**Interview Tool S1**

**Topics and the corresponding open-ended guiding questions for the three informant groups**

| Journalists | |
| --- | --- |
| Topic | **Corresponding Open Ended Interview Questions** |
| Role of media in policymaking, advocacy and shaping public opinion | 1. In your opinion, what is the role of media in influencing policymaking? Do you usually measure the impact of news report on policy decisions? If yes how? Give us examples of stories that led to an impact. 2. At what stage do you usually cover stories of public policies? 3. Do you usually get feedback from policymakers on your stories? If no, why in your opinion policymakers do not follow up and give feedback on health stories? 4. Do media investigate certain policy decisions, look at how they are made and explore the role of evidence in these decisions? If no, why not? 5. What is the role of media in shaping public opinion and influencing individual health behavior? |
| Factors influencing health reporting and the use of evidence in health journalism | 1. Why some research topics get the attention of the media while others do not? How do you select and identify you stories? What made a story newsworthy?   What are the factors that influence the selection of specific stories (topics), researchers and scientists?   1. Do you have enough time to report on health news and access research? How is timing affecting coverage, accuracy and quality of health reporting? 2. How is competition (getting a scoop) affecting the quality of health reporting? 3. Who are your target audience? Does having a target audience in your mind affect the way you are framing your story? 4. What are the barriers to access and incorporate evidence into health journalism? 5. If you access research do you understand it? Does the language of research negatively impact evidence-based health reporting? In what format do you prefer research to be catered to media? |
| Role of evidence in health journalism | 1. What is your main source of health information? What do you consider as a credible source of information? 2. What’s the role of evidence in health journalism in Lebanon? 3. How do you know about a certain research? How and where do you access research evidence? Are you aware of certain databases? What are the journals that do you access? How do you identify the database? 4. How is your network with researchers (personal relationship, interaction) facilitating access to information? |
| Quality of health reporting | 1. Do you send your report to the source for verification? Why not? Do you think it is necessary? 2. How do you check on the accuracy of information and claims? How do you decide if the claim is investigative or not? How do you verify claims? What is the role of investigative journalism in Lebanon? Do you check claims and opinions against evidence? 3. Is there a specific set of criteria to assist you in reporting research evidence? 4. Is there a specific set of criteria for reporting international studies? 5. How do you perceive ethical issues in your work? |
| Strategies to improve the quality of health reporting and incorporate more evidence into health journalism | 1. Were you trained on reporting health news? If yes, what type of training? What areas are you interested to have training in? 2. What can be done to make access to research evidence easy and fast? 3. In your opinion, how can we improve the quality of health reporting and incorporate more robust evidence into journalism? |
| Policymakers | |
| Role of media in policymaking | 1. How do you perceive the role of media in influencing policymaking and getting the attention of policymakers to a certain problem? 2. Do the media influence your perception on the importance of certain issue? Give us an example of a health story that triggered you to take a decision, change a decision, or engage in policy debate? 3. Do the media play a role in making policymakers accountable for their decisions and for misusing or abusing evidence? Can the media be considered as an effective tool for accountability in Lebanon? 4. In your opinion, how can the media exert greater influence on policymakers? |
| Quality of health reporting | 1. Do you perceive the media as an accurate source of information? If not why? 2. Would evidence-based health reporting makes of the media a more credible source of information? |
| Role of evidence in health journalism | 1. What is the role of evidence in health journalism in Lebanon? 2. What role do the media play in linking research and policymaking worlds? In other words, what role do the media play in getting research evidence to policymakers? |
| Researchers | |
| Role of media in policymaking and shaping public opinion | 1. What is the role of research in influencing policymaking? What are the strategies you use to make your research reach policymakers? What role the media plays in this? 2. How do you perceive the role of media in influencing policies? 3. What is the role of media in shaping public opinion? |
| Factors influencing the use of evidence in health journalism and the quality of health reporting | 1. Do you think it is important to report health news and disseminate research in media? Why? 2. What are the barriers that may constrain the interaction and engagement with media? 3. Do you provide the journalists with additional references for their reports (other than the interview)? |
| Strategies to improve the quality of health reporting and incorporate more evidence into health journalism | 1. In your opinion how can researchers/scientists effectively communicate with media? What are the strategies to increase interaction between researchers/scientists and media and incorporate more evidence into health journalism? |
| Investigating the role of evidence in health journalism | 1. Do you usually disseminate your research through media? How do you connect with media (interviews, press releases)? Who initiate the contact? |
